# Prevalence, determinants, and characteristics of extemporaneous compounding in Jordanian pharmacies

**DOI:** 10.1186/s12913-019-4684-y

**Published:** 2019-11-08

**Authors:** Hatim S. AlKhatib, Sara Jalouqa, Nour Maraqa, Anna Ratka, Eman Elayeh, Suha Al Muhaissen

**Affiliations:** 1 Department of Pharmaceutics and Pharmaceutical Technology, School of Pharmacy, The University of Jordan, Queen Rania Street, Amman, 11942 Jordan; 2 Wegmans School of Pharmacy, St. John Fisher College, Rochester, NY USA; 3 Department of Biopharmaceutics and Clinical Pharmacy, School of Pharmacy, The University of Jordan, Amman, Jordan

**Keywords:** Extemporaneous compounding, Pharmacy practice, Dosage forms, Community pharmacy services, Hospital pharmacy, Pharmacists, Prescriptions, Pharmaceutical care, Jordan

## Abstract

**Background:**

Pharmaceutical compounding is an essential component in pharmacy practice allowing pharmacists to provide dosage forms or strengths that are commercially unavailable. Medications compounded for patient-specific needs contribute to personalized medicine. Extemporaneous compounding provided by pharmacies overcomes the market shortage of these therapeutic products. The aim of this study is to investigate and characterize the prevalence, characteristics, and determinants of extemporaneous compounding in Jordanian pharmacies.

**Methods:**

This study was based on a cross-sectional questionnaire and included 431 randomly selected pharmacies in the twelve governorates of the country. Data were collected via face to face interviews of pharmacists who voluntarily and verbally responded to the questions.

**Results:**

Results revealed that 223 (51.7%) of the surveyed pharmacies practiced extemporaneous compounding. The main reason for not providing extemporaneous compounding services was lack of prescription orders for compounded preparations (53.8%). The second reason was lack of the equipment and supplies necessary for compounding (24.4%). Extemporaneous compounding prescriptions were mainly issued by dermatologists (98.2%); dermatological indication was the most common of all extemporaneous compounded prescriptions. The main reason for requesting compounded medications was the lack of a commercially available product (87.9%). The vast majority of the compounded dosage forms were creams (99.6) and ointments (91.5), followed by solutions (23.3%). Only 5 (2.2%) of the studied compounding pharmacies prepared sterile products. The major sources for compounding protocols were the physician order (94.2%), and ‘in-house’ protocols (44.8%). However, the main resource for estimating compounded medications expiration date was information based on pharmacist’s experience (57.8%) and the physician’s order (53.4%).

**Conclusions:**

Extemporaneous compounding is a common element of pharmaceutical care. Topical preparations are the most commonly compounded products. Finding from this study suggest that there is a need for standardizing the compounded product formularies, product quality testing, and improving the consistency in estimation of an expiration date of compounded products.

## Background

Extemporaneous compounding refers to the practice of the preparation of a therapeutic product by mixing and combining pharmaceuticals agents for an individual patient in response to an identified need [1]. Extemporaneous compounding was traditionally used to fill most of the prescription orders submitted to pharmacists until the advent of pharmaceutical industry in the second half of the nineteenth century [2].

Nevertheless, extemporaneous compounding is still a relevant pharmaceutical service provided by many pharmacies worldwide [3–8]. The continued need for extemporaneous compounding in the age of pharmaceutical manufacturing is driven by the individualized patient care focusing on the therapeutic needs of patients with rare diseases [9], pediatric patients [10–12] and patients with requirements for special dosage forms, individualized dosing or active ingredients combination that are not provided commercially [1, 2, 6]. Moreover, from a professional point of view, compounding is a very unique attribute of a pharmacy practice that reflects on the professional status of pharmacists’ provision for patient-specific healthcare needs [5].

Application of quality standards in extemporaneous compounding is still a major concern for regulatory agencies since poor quality of compounding practices can result in life-threatening contamination or in products that don’t possess the required strength, quality, and purity [13]. In the United States, the process of extemporaneous compounding of sterile and nonsterile preparations in pharmacies, which are under the authority of the state boards of pharmacy, has been regulated, to varying extents, by the United States Pharmacopeia (USP) chapters <795> *Pharmaceutical Compounding – Nonsterile Preparations* and <797> *Pharmaceutical Compounding – Sterile Preparations* [14]. However, the safety and efficacy of compounded products are not clinically evaluated [15]. Extemporaneous compounding is performed in a considerable number of Jordanian pharmacies; however, the practice has never been assessed and characterized by any previous study. This study was designed to investigate the prevalence, characteristics, and determinants of extemporaneous compounding in Jordan. The findings from this study will improve the understanding of current extemporaneous compounding practices and provide information to guide efforts on developing the regulatory framework, educational curricula, and professional training needed to ensure safety and quality of compounded products and pharmaceutical care of patients.

## Methods

### Data collection

Data for this project were collected using a cross-sectional descriptive questionnaire. The questionnaire was constructed based on information collected from intense review of published relevant literature [1, 6, 15, 16]. A 5-section questionnaire was developed to obtain data from pharmacies to assess prevalence, frequency, and determinants of extemporaneous compounding (Additional file 1). The first section had thirteen items related to demographics of pharmacies and pharmacists’ and descriptive data including: pharmacy name, address, and type, pharmacist gender, age, qualifications, and position, and the number of the pharmacy staff members; training provided by the pharmacy, and provision of compounding services. The second section described prescriptions characteristics and reasons for compounding. The third section assessed the indications, types, and dosage forms of compounded medications. The fourth section asked about tools and equipment available for compounding. The fifth section assessed the formulation properties; resources for compounding protocols and estimation of expiration date for compounded medications.

Administration of this questionnaire was based on a personal interview with the pharmacist on duty; the data collector asked the pharmacist the questions and filled the questionnaire. Four senior pharmacy students performed the interviews with the participants. All interviewers were trained by the principal investigator (PI) and two of the co-investigators. The training focused on communication skills with the pharmacy staff and on correct administration of the questionnaire. Moreover, the questionnaire was supplemented with a protocol with instructions. The interviewers were instructed to contact the PI in case of any uncertainty to ensure consistency in administration of the questionnaire.

The questionnaire was distributed to pharmacists from 431 randomly selected community and hospital pharmacies located in cities in the 12 governorates of Jordan. There were no exclusion criteria. The population frame was obtained as a list of all operating pharmacies (community and hospital) in the twelve governorates of Jordan. The list was provided by the Jordan Pharmacy Syndicate. Data were collected in February, 2018. Participating pharmacists were requested to sign an informed consent before responding to the questionnaire.

The data collection tool was evaluated by an expert in the field of pharmacy practice and piloting. A pilot test of the 26-item questionnaire was conducted in 30 pharmacies that were randomly-selected. The results from the pilot study were evaluated and used to modify the data collection tool before starting the main data collection period. Statistical confirmation of validity and reliability was achieved through the calculation of Cronbach’ Alpha value which was found to be 0.907 indicating consistency of the tool. Sampling adequacy was also confirmed using Principal Components Analysis (PCA) with Kaiser-Meyer-Olkin (KMO) equal to 0.841 and a significant Bartlett’s Test (*p* = 0.000).

To minimize social desirability bias, assurance was given to interviewees that discussions would be confidential and their responses would be anonymized. Collected data was stored with the corresponding author and further analysis was done anonymously.

### Statistical evaluation

The sample size was calculated using compounded medications prevalence values (P) described in Buurma et al. [16] and Zaid et al. [6], Z_α_ = 1.96, and d = 0.05. This calculation showed that 50 to 308 pharmacies were required to ensure reliable sample size to describe the compounded medication frequency and characteristics.

Categorical data were presented as frequency and percentages, while continuous data were presented as median and interquartile ranges.

Bivariate analysis was used to test the differences among the variables that affect the compounding of extemporaneous drug preparations. Chi-square or Mann-Whitney tests were used to detect differences between different determinants. Logistic regression was used to evaluate the strength of association of factors that significantly determine the characteristics of compounding in the bivariate analysis. All hypothesis testing was two-sided. A *P*-value of < 0.05 was considered significant. Data analysis was performed using SPSS® 23.0 (IBM, Chicago, IL).

## Results

The data collection team approached 444 community and hospital pharmacies. Invitation to participate in this study was accepted by 431 (97.1%) pharmacies. A total of 408 community pharmacies (18.1% of all Jordanian community pharmacies [17]) and 23 hospital pharmacies (21.7% of all Jordanian hospitals [18]) provided responses to the study questionnaire.

Demographics and basic characteristics of the participating pharmacies are presented in Table 1 and Table 2. The vast majority of the participating pharmacies were community pharmacies that were not part of a chain, and half of these pharmacies were run by two pharmacists. The pharmacy staff has one pharmacy assistant and one staff personnel in 130 (30.2%) and 171 (39.7%) of pharmacies, respectively. More than 400 (93%) of the interviewed staff were pharmacists, and 375 (87%) of them had the bachelor university degree, while the rest of pharmacists have a master or PhD degree as shown in Table 2. the median daily number of prescription received by pharmacies (with or without compounding orders) was 20 (IQR:10–30) while the median daily number of prescriptions containing at least one compounding order was 1.5 (IQR: 1–4.8). Table 2 also shows factors that the pharmacy staff consider in deciding whether to compound prescribed medication. The main reason for requesting compounded medication is the unavailability of the required medication (196, 87.9%), followed by lack of the needed dose among commercially available products (152, 68.2%). The main reason for not performing compounding was the absence of compounding orders in (232, 53.8%). The lack of equipment, tools, facilities, and resources required for compounding of prescriptions was reported by 24.4% of pharmacies as a reason for not compounding.

**Table 1:** Demographic characteristics of pharmacies participating in the Study

Characteristic	Number (%)
Pharmacies invited to participate in study	444(100)
Pharmacies that participated in study	431(97.1)
Pharmacy Type	
Hospital	23 (5.3)
Community	408 (94.7)
Chain	114 (27.9)
Independent	294 (72.1)
Pharmacist Gender (*N* = 431)	
Female	235 (54.5)
Male	196 (45.5)
Pharmacist Age, years	
23–30	220 (51.0)
30–40	105 (24.4)
40 +	106 (24.6)
Personnel	
Pharmacist	403 (93.5)
Registered Pharmacy Assistant	28 (6.5)
Pharmacists’ Qualification	
Doctorate Degree	3 (0.7)
Master Degree	15 (3.5)
Bachelor Degree	375 (87)
Diploma Degree	38 (8.8)
Number of Pharmacists per Pharmacy	
1	84 (19.5)
2	216 (50.1)
3	78 (18.1)
4	29 (6.7)
≥5	24 (5.6)
Number of Assistants per Pharmacy	
0	236 (54.8)
1	130 (30.2)
2	54 (12.5)
≥3	11 (2.5)
Number of Personnel per Pharmacy	
0	223 (51.7)
1	171 (39.7)
2	25 (5.8)
≥3	12 (2.8)
Plan to Start Compounding Services (*N* = 208)	
Yes	66 (31.7)
No	142 (68.3)
Training on Compounding Provided in Pharmacy	
For Pharmacists	235 (54.5)
For Registered Assistants	109 (25.3)

**Table 2:** Characteristics of Participating Pharmacies Performing Extemporaneous Compounding

Parameter	N (%)	Median (IQR)
Perform Compounding		
Yes	223 (51.7)	
Hospital	3(1.3)	
Community	220 (98.7)	
Yes	208 (48.3)	
Hospital	20 (9.6)	
Community	188 (90.4)	
Average Number of Total Prescriptions/day (*N*=382)		20 (10–30)
Average Number of Prescriptions per day for Compounded Orders* (*N*=16)^a^		1.5 (1–4.8)
Average Number of Prescriptions per week for Compounding Orders* (*N*=42)^a^		2 (1–3)
Average Number of Prescriptions per month for Compounding Orders* (*N*=164)^a^		2 (1–4)
Total Number of Years of Providing Compounding (*N*=220)		10 (4–18)
Reasons for Providing Compounding Services (*N* = 223)^b^		
Medication is not commercially available	196 (87.9)	
Dosage Form not commercially available	51 (22.9)	
Needed Dose not commercially available	152 (68.2)	
Stability of the desired product	6 (2.7)	
Improve adherence to medications	102 (45.7)	
Other	1 (0.4)	
Reasons for Not Providing Compounding Services (*N* = 431)^b^		
No prescriptions for compounded preparations	232 (53.8)	
Compounding require a lot of time (No time)	22 (5.1)	
No equipment or/and supplies	105 (24.4)	
Pharmacy staff lack skills/training on compounding	15 (3.5)	
There are no regulations	9 (2.1)	
Lack of trust on compounded medications	23 (5.3)	
Compounding of medications is too expensive	14 (3.2)	
Compounding of medications is too difficult	32 (7.4)	
Compounded medications final cost is too high	11 (2.6)	
Others	5 (1.1)	

^a^Pharmacies were asked initially about average daily number of prescriptions that contain compounding orders, if not applicable asked about average weekly number, if not applicable about average monthly number

^b^Data do not sum up to 100% as multiple responses/selections were allowed

Abbreviations; *IQR* Interquartile Range

Table 3 shows data comparing pharmacies that perform compounding and pharmacies that do not compound. Being an independently-owned or chain pharmacy had no effect on its practice of compounding. The presence of a pharmacy technician (two-year diploma) as a part of the pharmacy staff did not affect the compounding practice of pharmacies. Significant differences were observed between hospital and community pharmacies in terms of compounding; community pharmacies were more involved in compounding. Pharmacies that prepared compounded medications had significantly higher number of pharmacists and personnel, and received, on average, a higher number of prescriptions per day.

**Table 3:** Comparison of key characteristics of compounding and non-compounding pharmacies

Determinant			*p-value*
Perform Compounding	Yes (*N* = 223)	No (*N* = 208)	
	N (%)		
Pharmacy Type: Hospital	3 (13)	20 (87)	0.000*
Community	220 (53.9)	188 (46.1)	
Chain	69 (60.5)	45 (39.5)	0.623*
Independent	151 (51.4)	143 (48.6)	
	Mean ±SD		
Number of Pharmacists	2.7 ±1.0	2.1 ± 1.8	0.000^¥^
Number of Assistant	0.64 ± 0.9	0.69 ± 0.9	0.600^¥^
Number of Personnel	0.7 ± 0.8	0.5 ± 0.7	0.022^¥^
Average Daily Number of All Received Prescriptions	20 (10-40)^#^	15 (6-27.8^#^	0.000^¥^

^*^p values were calculated using Chi square test

^¥^*p* values were calculated using Mann Whitney test

^#^median (interquartile range)

Factors that showed significant baseline differences between compounding and non-compounding pharmacies were entered into model testing using logistic regression to test for significant association with the decision of the pharmacy to provide compounding service. As shown in Table 4, the model was significant (OR = 1.47; *p* = 0.000). The number of pharmacists (OR = 1.82; *p* = 0.000), the number of personnel (OR = 1.09; *p* = 0.043) and the average number of daily prescriptions received by the pharmacy (OR = 1.02; *p* = 0.002) were significantly associated with this service provision. While the type of pharmacy being hospital or community pharmacy and being part of a chain or not showed no association with compounding services provision.

**Table 4:** Analysis of Factors that Affect the Decision of Pharmacy to Perform Compounding Results Using Stepwise Logistic Regression Analysis

Determinant	Regression		
	OR	95% CI	*p*-value
Model	1.47		0.000
Number of Pharmacists	1.82	1.35–2.45	0.000
Number of Personnel	1.09	0.72–1.65	0.043
Average Daily Number of All Prescriptions	1.02	1.01–1.04	0.002

Table 5 presents the types of compounded medications and compounding services. Dermatologist were the main prescriber specialty group requesting compounded preparations (219, 98.2%). Among the compounded dosage forms, 222 (99.6%) and 204 (91.5%), were creams and ointments, respectively, and 52 (23.3%) were solutions. Only 5 (2.2%) of the participating pharmacies that performed compounding prepared sterile products, with only 3 (1.3%) of compounding pharmacies making sterile eye drops (Table 5).

**Table 5:** Compounded Products and Services Provided by Compounding Pharmacies

Parameter	Number (%)
Specialty of Compounding Prescriber (*N* = 223)^a^	
Dermatology	219 (98.2)
Pediatrics	41 (18.4)
ENT	24 (10.8)
Gastroenterology	24 (10.8)
Radiology	11 (4.9)
Other	37 (16.6)
Compounded Dosage Forms (*N* = 223)^a^	
Tablets	0
Capsules	0
Suppository	1 (0.4)
Ointment	204 (91.5)
Cream	222 (99.6)
Solution	52(23.3)
Suspension	10 (4.5)
Syrup	2 (0.9)
Paste	9 (4)
Powder	3 (1.3)
Compounded Sterile Products (*N* = 223)	
Yes	5 (2.2)
No	218 (97.8)
Types of Compounded Sterile Products (*N* = 223)	
Eye Drops	3 (1.3)
Electrolytes	1 (0.4)
Total Parenteral Nutrition	2 (0.9)
Chemotherapy	2 (0.9)
Equipment and Tools Used for Compounding Availability (*N* = 431)^a^	
Mortars	214 (49.7)
Pestles	213 (49.4)
Capsule Machines	1 (0.2)
Ointment Mill	14 (3.2)
Tablet Machines	0
Suppositories Molds	7 (1.6)
Beaker	131 (30.4)
Funnel	125 (29)
Spatula	213 (49.4)
Scale	188 (43.6)
Flask	118 (27.4)
Water Bath	31 (7.2)
Measuring Cylinder	114 (26.5)
Glass Rod	137 (31.8)
Ointment Slab	60 (13.9)
Sources for protocols of Compounded Medications (*N* = 223)^a^	
Published Literature	5 (2.2)
USP	9 (4)
BP	25 (11.2)
Self-Reports	100 (44.8)
Other Pharmacies or Hospitals	11 (4.9)
Prescriber/Physician	210 (94.2)
Provision of Expiration Date on Compounded Medications (*N* = 431)	
Yes	156 (70)
No	67 (30)
Sources for Estimation of Expiration Date for Compounded Medication (*N* = 223)^a^	
Published Literature	8 (3.6)
USP	6 (2.7)
BP	16 (7.2)
Self-Reports	129 (57.8)
Other Pharmacies or Hospitals	7 (3.1)
Prescriber/Physician	119 (53.4)
Label Provided on Compounded Medication (*N* = 223)	
Yes	217 (97.3)
No	6 (2.7)
References for Information Provided on the Label of Compounded Medication (*N* = 223)	
Published Literature	0
USP	36 (16.1)
BP	11 (4.9)
Pharmacist’s Experience	149 (66.8)
Prescriber/Physician	146 (65.5)
Records for Compounded Medications (*N* = 431)	
Yes	54 (24.2)
No	377 (75.8)
Type of Record/Log-book (*N* = 54)	
Electronic	20 (37)
Hand-written	34 (63)
esting of Quality of Compounded Medications (*N* = 223)	0
Most Frequently Used Expiration Date, Weeks (*N* = 223)	4 (0.9)

^a^ Data do not sum up to 100% as multiple responses/selections were allowed

About half of pharmacies have mortars, pestles, spatula, and scales, and almost one third of pharmacies have funnels, flasks, beakers, measuring cylinders, and glass rods (Table 5).

The main source of references for compounding protocols were the physician’s order in 210 (94.2%) of the cases, and protocols developed by the pharmacy in 100 (44.8%) of the respondent pharmacies. The main source for estimation of the expiration date for compounded medications was based on pharmacist’s experience (129, 57.8%) and physician’s order (119, 53.4%). In 67 (30%) of the compounding pharmacies, expiration date was not provided for compounded medications but 217 (97.3%) of these pharmacies attach label to their compounded products. The expiration date included on the label was determined based on either in-house practice or physician’s order in 149 (66.8%) and 146 (65.5%) of the compounding pharmacies, respectively.

In 377 (75.8%) of the compounding pharmacies, no compounding records were kept, and 34 (63%) of these records were hand-written. No pharmacy performed quality control tests of the compounded products.

## Discussion

The current study addressed the prevalence, determinants, and characteristics of extemporaneous compounding and compounded products in both community and hospital pharmacies in Jordan. The present study is the first report in Jordan and one of few in the world that present the baseline data on extemporaneous compounding. Several important outcomes that emerged from this study can be used in the improvement of pharmacy practice.

The high prevalence of the compounding practice in community pharmacies emphasizes the need of proper education and training of pharmacy students in extemporaneous compounding. There is also a clear need to incorporate extemporaneous compounding in professional development and continued education programs offered by the Jordanian Pharmacy Syndicate and the Jordanian Pharmacy Board. Results from this study also show the critical need for implementation of quality testing of compounded products and standardized regulations to ensure safe compounding practices.

Data from this study show that 52% of Jordanian pharmacies perform compounding which is less than the percentage (add this percentage) reported in USA [3, 19], The percentage of prescriptions received per day that contain at least one compounding order is higher than those reported in literature [3, 6, 16, 19]. These differences may be attributed to the fact that this study involved all types of Jordanian pharmacies (large and small, and hospital and community pharmacies). Buurma and colleagues (2003), reported a decline in Dutch compounding pharmacies and a rise of specialized firms that compound and promote those preparations that are most broadly needed [16].

Several factors can play a role in the pharmacy’s decision to offer compounding services. These factors include the availability of the staff (both pharmacists and personnel) and the number of all prescriptions received and filled by the pharmacy. Pharmacies with a higher number of pharmacists reported making more compounded preparations. The time and efforts required to develop and manage extemporaneous compounding service are critical factors that influence the prevalence of compounding in Jordanian pharmacies. This issue has been addressed in Dutch community pharmacies by preparing a large part of compounded medications from semi-manufactured or almost finished products provided by specialized firms. Such products require minimal work to be finished at the pharmacy [16].

As would be expected, the number of all prescriptions filled by the pharmacy correlates directly with the compounding services. However, some pharmacies with a long tradition in providing compounding services were observed to have a disproportionately higher number of compounding order to their overall number of prescriptions filled daily.

Dermatologists were reported to be the prescribers of compounding orders by 98.2% of the compounding pharmacies in Jordan. They were ahead of other specialties by a large margin. This is in agreement with data from previously reported study on top indications for extemporaneously compounded products [16] and could be related to the observation that creams and ointments were the most commonly compounded dosage forms by compounding pharmacies.

The fact that community pharmacies were more involved extemporaneous compounding could be understood in the context that most of the compounded dosage forms are creams and ointments and the main prescriber specialty group was “Dermatologists”. Most health complaints addressed by dermatologists do not require hospitalization and prescriptions are often filled by community rather than hospital pharmacies.

Pediatricians were the second highest specialists in terms of requesting compounded products with 18.4% of the compounding pharmacies reporting receiving orders for compounding from pediatricians. Buurma and colleagues reported that patients less than 12 years old received more extemporaneously prepared medications than other age groups [16].

Solutions were the third most commonly compounded dosage form (23.3%) in Jordanian compounding pharmacies.

Previously reported studies showed that patient-centered pharmaceutical care is the main reason for pharmacists to provide compounded medications [3, 6]. Pharmaceutical care should address all patients needs by ensuring the availability of all needed medications, including ingredients combinations, dosage forms, and doses that are patient-specific. Individualized patient care results in improved patient’s adherence to medications and consequently better therapeutic outcomes and quality of life.

As shown in this study, the main reasons for requesting and providing compounding services is the unavailability of the required medications or a specific dosage form (87.9%) and the unavailability of the required dose (68.2%). An earlier study has linked the reasons for compounding with the specialty of the prescriber with dermatologists being reported to order combinations that are usually unavailable commercially and pediatricians requiring patients-tailored dosage strengths [16].

The majority of pharmacies participating in this study stated lack of prescriptions for extemporaneously compounded products as a key reason for not providing compounding services. Zaid et al. [6] and McPherson et al. [3] also reported that that the main reason for pharmacists being non-compounders is not receiving prescriptions. Surprisingly, the second reported reason for not compounding was the lack of equipment and supplies. This is unexpected because by the law, each licensed pharmacy is required to have compounding tools and equipment. Interestingly, required time, cost, and difficulty of preparing these medications did not have impact on the pharmacy’s decision to provide compounding services.

In general, sterile compounding was not performed in pharmacies participating in this study. This can be explained by the fact that compounding of sterile products is expensive because it requires sterile environment, specific procedures and equipment. Improper preparation of sterile compounded preparations may easily lead to contaminated product [6].

Buurma and colleagues reported that in 42% of the cases, pharmacies did not use a standardized or semi-standardized protocol [16]. The current study showed the lack of standardized procedures for compounding with vast majority of the respondents stating that the physician order is the source of the procedure while 44.8% reported the use of procedures developed in the pharmacy for compounding different products. The same resources were reported to be relied upon for estimation of expiration date for compounded medicines and labeling of the compounded products.

Moreover, the study uncovers the lack of documentation and quality assurance of the compounded product, although 24.2% of pharmacies keep records for their products and considerable number of pharmacists seeks patients’ feedback on their products. Despite of all of this, there is a need for agreed on protocols that cover procedures, labelling including expiry date, and quality control tests that should be followed and guaranteed during extemporaneous compounding to assure effectiveness and quality of the final product and improve the physicians as well as patients’ confidence in these products.

The main limitation of this study is the difference in the number of community (408) and hospital pharmacies (23) included in the study. However, it is worth noting that we tried to include a similar proportion of community (18.1%) and hospital (21.7%) pharmacies of all those operating in Jordan. Another limitation is the fact that this study did not cover the compounding practice from the perspective of prescribers and patients. This includes the study of drivers for prescribing compounded products and the patients’ experience with the compounding services offered by pharmacies. We are attempting to cover this aspect in a future study.

The data presented in this study was presented to and discussed with faculty members at the School of Pharmacy, University of Jordan in order to modify curriculum to better cover procedures, labelling including expiry date, and quality control tests that should be followed and guaranteed during extemporaneous compounding to assure effectiveness and quality of the final product and improve the physicians as well as patients’ confidence in these products. In addition, a plan was developed for offering a continuing education program to practicing pharmacists in collaboration with the Jordanian Pharmacists Syndicate in the area of best practices in extemporaneous compounding.

## Conclusion

Extemporaneous compounding is a widely spread pharmaceutical practice among community pharmacies in Jordan. Ointments and creams are the most commonly prescribed and compounded extemporaneous products. A higher number of pharmacists, pharmacy personnel and the total number of prescriptions were significantly associated with a higher number of extemporaneous products compounded. The lack of standardized procedures for preparing extemporaneous products, scientific basis for determination of expiration date of compounded products, and lack of quality control testing prompts an action.

## Supplementary information

**Additional file 1.** Extemporaneous Compounding In Jordanian Pharmacies Survey .

## Data Availability

The datasets used and analyzed during the current study are available from the corresponding author on reasonable request.

## Abbreviations

IRB: Institutional Review Board
KMO: Kaiser-Meyer-Olkin
P: prevalence values
PCA: Principal Components Analysis
PI: principal investigator
SP-UJ: School of Pharmacy – University of Jordan
USP: United States Pharmacopeia
