# Supplementary material for: Prevalence, determinants, and characteristics of extemporaneous compounding in Jordanian pharmacies
Source: BMC Health Serv Res. 2019 Nov 8;19:816. doi: 10.1186/s12913-019-4684-y (PMC6842227; doi:10.1186/s12913-019-4684-y)
Supplement: Supplementary file 1 — Additional file 1. Extemporaneous Compounding In Jordanian Pharmacies Survey . [file 12913_2019_4684_MOESM1_ESM.docx]

# **Appendix I**

# Thank you for agreeing to take part in this research. This study aims to provide information about extemporaneous compounding (the preparation of medications in the pharmacy) in pharmacies in Jordan. Note that the information you give will be confidential and used for scientific research.

**Extemporaneous Compounding In Jordanian Pharmacies Survey**

# Please tick here to indicate your informed consent to participate in this study

# ***Part A. Demographic information***

A1. Pharmacy name: ……………………………………………………………………………………………………….

A2. Address: …………………………………………………………………………………………………………………….

A3. Pharmacy Type: Hospital Community

A4. Pharmacy Sub-type: Chain Non-chain

A5. Pharmacist name: …………………………………………………………………………… (Not obligatory)

A6. Pharmacist gender: Male Female

A7. Pharmacist age:  20-25  26-30  31-35  36-40  >41 years

A8. Pharmacist is:  Senior pharmacist

Community pharmacist

Registered assistant

Intern

Others ………………………………………………………

A9. Pharmacist qualifications: Doctorate degree

Master degree

Bachelor degree

Others………………………………………………………

A10. Pharmacist training on preparing extemporaneous compounds:  Yes  No

A11. Number of Pharmacists: ……………

A12.Number of Assistants: ……………

A13.Number of personnel: ……………

# ***Part B. Prescriptions.***

B1. Average daily number of prescriptions: ……………

B2. Average daily number of prescriptions that include compounding: ………………………….

If Not Applicable

Average weekly number of prescriptions that include compounding: ………………………

If Not Applicable

Average monthly number of prescriptions that include compounding: …………………….

B3. Prescriber specialty:

GP dermatologist ENT Physician  pediatrician other: ………………

B4. Prescription repetition:  First time prescription

Second time prescription

More than two times prescribed

Other ………………………………………………………………………………

B5. The reason for compounding:

The drug is not commercially available

Special dosage form is required

Off-label use of a commercially available medication

Allergy for an ingredient in a commercially available medication

Compliance (bitter taste)

Others …………………………………………………………………………………………………………

B6. Reasons for not providing compounding services:

I do not receive prescriptions that require compounding

I do not have enough time

The required equipment or supplies are not available in my pharmacy

Either my pharmacy staff or I lack appropriate training

There are no regulations to ensure good quality and safety

There is no trust in compounded medications

It is too expensive or difficult to maintain compounding services

Final cost is high

Others …………………………………………………………………………………………………………………..

# ***Part C. Medications.***

C1. Drug indications:

Dermatological

Cardiovascular system

Nervous system

Alimentary tract and metabolism

Systemic hormonal preparations

Respiratory system

Musculo-skeletal system

Sensory organs

Others: …………………………………………………………………………………………………………

C2. Dosage Form:

Tablet

Ointment

Cream

Solution/Suspension

Powder

Others: …………………………………………………………………………………………….……………

# ***Part D: Formulation.***

D1. Protocol used in formulation:

Published literature

Pharmacopeia

USP

BP

Self-reports on in-house formulations

From other pharmacy / hospital

Other ……………………………………………………………………………………………………………

D2. Protocol on deciding the expiry date:

Published literature

Pharmacopeia

USP

BP

Self-reports on in-house formulations

From other pharmacy / hospital

Other ……………………………………………………………………………………………………………

D3. An estimation of the expiry date:

24 hours 1 week 2 weeks1 month >1month other: ………………

D4. The use of labeling:  Yes  No

D5. Reference on labeling:

According to published literature

According to personal experience

Other: ……………………………………………………………………………………………………………

D6. Record keeping (Logbook): Yes  No

Pharmacist Email*: _____________________________.

*If you want to be informed the results of the study.

***Thank You***
